# Isonitrogenous low-carbohydrate diet elicits specific changes in metabolic gene expression in the skeletal muscle of exercise-trained mice

**DOI:** 10.1371/journal.pone.0262875

**Published:** 2022-01-21

**Authors:** Hazuki Saito, Naoko Wada, Kaoruko Iida

**Affiliations:** 1 Department of Food and Nutrition Science, Graduate School of Humanities and Sciences, Ochanomizu University, Otsuka, Bunkyo, Tokyo, Japan; 2 The Institute for Human Life Innovation, Ochanomizu University, Otsuka, Bunkyo-ku, Tokyo, Japan; Poznan University of Physical Education, POLAND

## Abstract

With the renewed interest in low-carbohydrate diets (LCDs) in the sports field, a few animal studies have investigated their potential. However, most rodent studies have used an LCD containing low protein, which does not recapitulate a human LCD, and the muscle-specific adaptation in response to an LCD remains unclear. Therefore, we investigated the effects of two types of LCDs, both containing the same proportion of protein as a regular diet (isonitrogenous LCD; INLCD), on body composition, exercise performance, and metabolic fuel selection at the genetic level in the skeletal muscles of exercise-trained mice. Three groups of mice (n = 8 in each group), one fed a regular AIN-93G diet served as the control, and the others fed either of the two INLCDs containing 20% protein and 10% carbohydrate (INLCD-10%) or 20% protein and 1% carbohydrate (INLCD-1%) had a regular exercise load (5 times/week) for 12 weeks. Body weight and muscle mass did not decrease in either of the INLCD-fed groups, and the muscle glycogen levels and endurance capacity did not differ among the three groups. Only in the mice fed INLCD-1% did the plasma ketone concentration significantly increase, and gene expression related to glucose utilization significantly declined in the muscles. Both INLCD-1% and INLCD-10% consumption increased gene expression related to lipid utilization. These results suggest that, although INLCD treatment did not affect endurance capacity, it helped maintain muscle mass and glycogen content regardless of the glucose intake restrictions in trained mice. Moreover, an INLCD containing a low carbohydrate content might present an advantage by increasing lipid oxidation without ketosis and suppressing muscle glucose utilization.

## Introduction

It is generally accepted that adequate carbohydrate intake is one of the fundamental nutritional strategies to promote exercise performance. Despite that, there has been an interest in low-carbohydrate diets (LCDs) among athletes because of the metabolic adaptation to specific nutritional compositions of LCDs, such as enrichment with fat while restricting carbohydrates. From this point of view, research trials investigating the effect of an LCD on exercise capacity and detailed metabolic changes have been conducted in athletes and trained subjects from the 1980s to the early 2000s [1–5]. However, despite the positive aspects of an LCD, such as enhanced fat oxidation and reduced utilization of muscle glycogen during exercise, many of these studies have reported no obvious effects with regard to exercise performance [1–5] and, as a result, LCD seemingly failed to become of great interest among athletes originally.

In recent years, there has been an increasing interest in decreasing carbohydrate intake as one of the forms of dietary treatment for metabolic diseases, such as obesity and diabetes. Many studies have been conducted using an LCD, and many review articles have discussed the effectiveness of this type of diet as a dietary therapy [6–8]. Such an LCD, which strictly limits the intake of carbohydrates [6], was originally used as a precaution against seizures caused by intractable epilepsy, and is also called a ‘ketogenic diet’ because ketosis was observed in the patients consuming this diet. However, over the past 20 years, many studies have demonstrated the effectiveness of LCDs in decreasing the body weight of obese patients and improving blood parameters of diabetic patients, despite its high-fat content [9–12]. Consequently, a renewed interest in ketogenic LCDs—particularly in the purported benefits on body composition, as well as physical health and sports performance—has also emerged among athletes in the field of sports nutrition. More than 10 research trials have been performed in the last few years to assess the effects of LCDs on various physiological parameters and exercise performance in exercise-trained athletes (reviewed in Ref. [13, 14]), and the number of studies focusing on the effect of LCD on athletes shows arising trend [15–17].

As human trials are always restricted due to various reasons, several studies have also been conducted using animals that investigated the exercise capacity and the detailed metabolic changes associated with LCDs [18–21] to evaluate the potential of LCDs as a nutritional strategy for sports performance. However, we previously reported that sole LCD exposure versus pairing an LCD with exercise training had different impacts on energy substrate selection in organs with high energy demand [22]; thus, the findings obtained from sedentary animal models might not accurately reflect the biological phenomena that occur in exercise-trained subjects. There are also some problems with the dietary composition of the LCDs used in previous animal studies. While many human studies have adopted LCDs that contain up to 50 g of carbohydrates (about 5–10% of total energy) [13], rodent studies have frequently used an LCD that was extremely low in carbohydrates (less than 1% of total energy). In addition, the commercially available low carbohydrate ketogenic diet for rodents contain considerably less protein (less than 10% of total energy) compared to the regular rodent chows and a human LCD therapeutically used for metabolic diseases, which is typically formulated so that roughly 5% of the total energy intake were from carbohydrates, 20% from protein, and 75% from fat [23].

Here, we hypothesized that an LCD containing adequate protein would have a different impact on exercise capacity and muscle metabolism than those observed in previous animal studies which have reported a positive effect of LCDs on weight reduction or on exercise performance in trained animals. To support this hypothesis, we evaluated the effects of an LCD isonitrogenous to a regular diet on body composition, endurance capacity, and muscle metabolic gene expression by imposing long-term exposure to isonitrogenous LCDs on a set of mice given a regular exercise load. We also examined whether the limited glucose intake could potentially cause sparing of muscle glycogen during exercise in these mice. For this purpose, we tested two types of LCDs containing carbohydrates at different proportions (1% and 10%) of total energy intake.

## Materials and methods

### Experimental diets

An AIN-93G rodent diet (D10012G, RESEARCH DIETS Inc. NJ, USA), composed of 16% fat, 20% protein, and 64% carbohydrate (4.0 kcal/g of energy), was used for the regular diet as a control. The two different types of isonitrogenous LCDs (INLCDs) based on different proportions of carbohydrates were prepared as follows: 1) INLCD-10% composed of 69% fat, 20% protein, and 10% carbohydrate (6.1 kcal/g of energy), and 2) INLCD-1% composed of 78% fat, 20% protein, and 1% carbohydrate (6.6 kcal/g of energy). The ingredients were purchased from Oriental Yeast Co. (Tokyo, Japan), and the composition of each diet is shown in Table 1.

**Table 1:** Ingredient composition of the diets.

Ingredient	AIN-93G	INLCD-10%	INLCD-1%
Casein [g]	20	30.7	33.3
L-cystine [g]	0.3	0.5	0.5
Soybean oil [g]	7	4.6	5.6
Lard [g]	0	42.4	51.9
Corn starch [g]	39.75	13	0
Maltodextrin [g]	13.2	0	0
Sucrose [g]	10	0	0
Cellulose [g]	5	4.1	4.4
Vitamin mix ^a^ [g]	1	1	1
Choline bitartrate [g]	0.25	0.25	0.25
Mineral mix ^b^ [g]	3.5	3.5	3.5
Total [g]	100	100	100
Energy [kcal/100 g]	403	608.7	659.3
Protein [kcal/100g (% of energy)]	81.2 (20)	124.6 (20)	135.0 (20)
Fat [kcal/100 g (% of energy)]	63.0 (16)	422.6 (69)	516.8 (78)
Fatty acids, saturated [g/100g]	1.0	17.3	21.2
Fatty acids, monounsaturated [g /100g]	1.5	19.5	23.8
Fatty acids, polyunsaturated [g /100g]	3.9	6.7	8.2
Carbohydrate [kcal/100g (% of energy)]	258.8 (64)	61.5 (10)	7.5 (1)

^a^Containing 97.4% (wt/wt) sucrose.

^b^Containing 22.1% (wt/wt) sucrose.

The number of the percentage of energy in the parentheses are round off decimal places.

### Animals and rearing conditions

**Fig 1:**
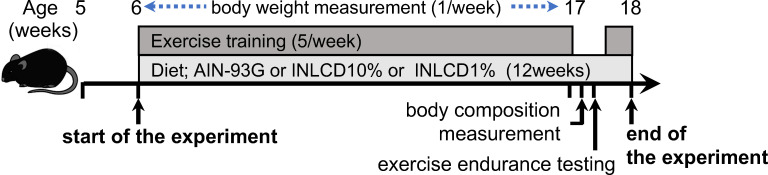
Timeline of the experimental protocol.

The experimental timeline is summarized in Fig 1. A total of 24 male C57BL/6J mice were obtained at five weeks of age (Japan SLC, Hamamatsu, Japan). After feeding the mice a regular diet for 1 week, the animals were randomly assigned to three groups: those fed the regular diet (Control; CON group), those fed INLCD-10% (INLCD-10 group), and those fed INLCD-1% (INLCD-1 group) (n = 8 for each group). Four mice were housed together in each cage and reared for 12 weeks. Animals were housed at room temperature (22±1°C), with a 12 h light-dark cycle and allowed free access to food and water. Food intake and body weight were both measured every week until 1 week before the experiment was terminated. At the end of the experimental period, the mice were fasted for 4 h (from 9 a.m. to 1 p.m.) and anesthetized with isoflurane to collect blood samples. After blood collection, the mice were euthanized by cervical dislocation under anesthesia and immediately dissected to collect tissue samples. All experiments were approved by the Ochanomizu University Animal Ethics Committee (approval number: 18015) and performed according to the “Guidelines for Proper Conduct of Animal Experiments” of the Science Council of Japan and the “Guide for the Care and Use of Laboratory Animals 8th ed.” published by the National Academies Press.

### Exercise protocol

From the time the experimental diets were implemented, all animals were exercise-trained at a moderate intensity for 5 days a week, on week days with weekends off, using a small animal treadmill (MK-680, Muromachi, Tokyo, Japan) set to 15 m/min, 0% slope, for 60 min, based on the protocols from our previous studies [22, 24]. At this setting, the exercise intensity reaches to around 70% VO_2_ max in adult mice [25] and we have confirmed in previous studies that this intensity is enough to induce an adaptation to the exercise in mice [22, 24]. This regular exercise training was terminated 1 day before the end of the experiment. The endurance capacity was analyzed in each mouse during week 11 of the experimental period as follows: mice ran on a treadmill starting at a speed of 10 m/min and 5° slope for 2 min, and every subsequent 2 min, the speed was increased by 2 m/min until the mice were exhausted. Exhaustion was defined as the inability to keep running despite mechanical prodding.

### Analysis of trunk fat mass

Trunk fat mass was assessed in each mouse using a micro-X-ray computed tomography (CT) system (R_mCT2, Rigaku, Tokyo, Japan) a week before the end of the experiment. The mice were fasted overnight and then temporarily anesthetized with isoflurane, placed in the system, and then the images were subsequently captured from the diaphragm to the inguinal region. The X-ray tube voltage, current, and field of view were set at 90 kV, 88 μA, and 60 mm, respectively according to the manufacturer’s instructions. The weight of total fat mass was calculated using fat analysis software (Rigaku Co., 24 Tokyo, Japan).

### Biochemical analysis of blood serum

The blood samples collected in 1.5-mL microcentrifuge tubes were centrifuged at 10,000 × *g* for 5 min in a microcentrifuge (Model 3500, Kubota, Tokyo, Japan), and the supernatant was transferred to a new tube and stored at −20°C for analysis. Blood glucose concentration was measured using a biochemical colorimetric assay kit, LabAssay^TM^ Glucose (Cat. #298–65701, Wako, Tokyo, Japan) following the manufacturer’s protocol. Non-esterified fatty acids (NEFA) and triglycerides were measured using LabAssay^TM^ NEFA (Cat. #294–63601, Wako, Tokyo, Japan) and LabAssay^TM^ Triglyceride (Cat. #290–63701, Wako, Tokyo, Japan) following the manufacturer’s protocols, respectively. The ketone body (3-hydroxybutyrate) concentration was measured using a ketone body assay kit, Ketone Test Sanwa (Cat. #877434, Sanwa Kagaku, Tokyo, Japan), following the enzymatic protocol. All the absorbance measurements were carried out using an Enspire^®^ Microplate Reader (Perkin-Elmer Japan, Tokyo, Japan).

### Quantification of glycogen in muscle tissue

Glycogen was purified and quantified using the classical Pflüger method [26] in which glycogen is firstly extracted from the muscle tissue by alcoholic precipitation after alkaline tissue hydrolysis [27] and then hydrolyzed to glucose for the quantification of the glycogen content as glucosyl units [28]. Briefly, around 100 mg of the gastrocnemius (GC) muscle tissue was dissolved in 30% KOH at 95°C for 5 minutes. Then, 2% Na_2_SO_4_ and 66% ethanol were added, and the final solution was centrifuged (13000 × *g*, 5 min at 4°C) to obtain a pellet. The pellet was washed twice with 66% ethanol and then resuspended in 0.2 M acetate buffer (pH 4.5); amyloglucosidase (Sigma-Aldrich, Tokyo, Japan) was added, and the solution was incubated at 37°C for 30 min to hydrolyze the glycogen. Finally, the glucose content was measured using the LabAssay^TM^ Glucose kit (Wako, Tokyo, Japan), and the amount of glycogen per gram of tissue (wet weight) was then calculated.

### Gene expression analyses using real-time PCR

The tibialis anterior (TA) and the soleus (SOL) muscles collected from the mice were weighed and then cryopreserved at −80°C. Tissue RNA was extracted from TA and SOL muscles using Sepasol RNA I Super G (Nacalai, Kyoto, Japan) following the manufacturer’s protocol. First-strand cDNA was synthesized using the ReverTra qPCR Master Mix (TOYOBO, Osaka, Japan). SYBR Premix Ex Taq (Takara Bio, Shiga, Japan) was included in the real-time PCR reaction, which was run on a Thermal Cycler Dice Real Time System (Takara Bio, Shiga, Japan). We assessed the expression levels of the following genes, which are representative genes related to energy substrates’ utilization: pyruvate kinase (*Pk)*, glucose transporter 4 (*Glut4*), and hexokinase 2 (*Hk2*) for glucose; cluster of differentiation 36 (*Cd36*), carnitine palmitoyltransferase1-b (*Cpt1b*), and medium-chain acyl-CoA dehydrogenase (*Acadm*) for fatty acids; monocarboxylic acid transporter 1 (*Mct1*), monocarboxylic acid transporter 4 (*Mct4*), and succinyl-CoA 3-oxoacid CoA transferase (*Scot*) for ketone bodies. Relative gene expression was determined with β-actin as the internal standard using the ΔΔCt method [29]. The primer sequences of each gene used in the analysis are shown in S1 Table.

### Statistical analyses

Results are presented as the mean ± standard error of the mean (SEM). Statistical analysis was conducted using SPSS statistics (version 24, IBM, Tokyo, Japan). In the analyses, a two-way repeated analysis of variance (ANOVA) was used to detect differences in the body weight of animals between the groups over time (multifactorial ANOVA). For the body weight at each time point and other data, the Kruskal-Wallis test followed by Dann-Bonferroni post hoc test (for nonnormally-distributed data set) or one-way ANOVA followed by Tukey’s post hoc test (for normally-distributed data set) was performed to determine differences among groups. Results were considered statistically significant at p < 0.05.

## Results

### Effects of INLCDs on body and lower leg muscle weight and trunk fat mass in exercise-trained mice

**Fig 2:**
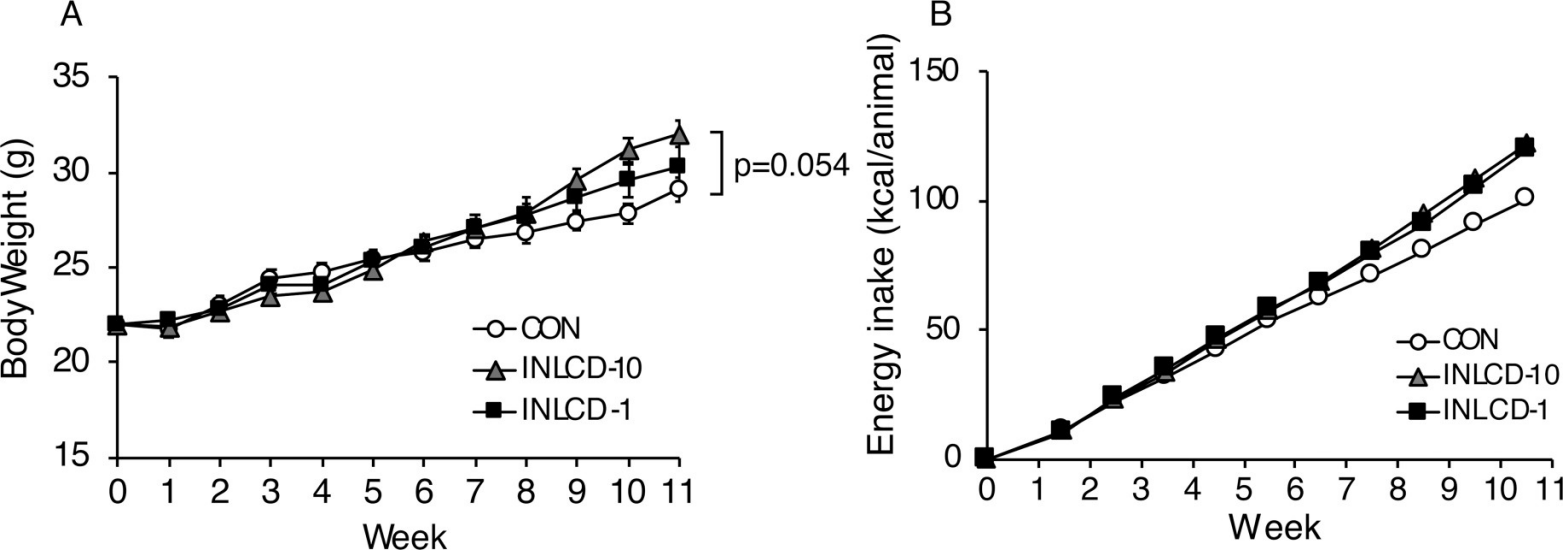
Effects of INLCD exposure on body weight and energy intake in exercise-trained mice. Mice were divided into three groups: those fed the control diet (CON), those fed an INLCD-10% (INLCD-10), and those fed an INLCD-1% (INLCD-1), and then reared for 12 weeks. Body weight (A) and total energy intake (kcal/mouse) (B) of mice during the first 11 weeks of the study are shown. Data are presented as means ± SEM, n = 8 per group. *, p < 0.05; **, p < 0.01 vs. CON.

At the beginning of the experiment, average body weight of mice was 22.0 g. During the experimental period, all mice gained weight since they were growing at their natural pace, and there was no significant difference in body weight between the groups until 8 weeks after starting the study (Fig 2A). However, body weight in the INLCD-10 group increased more than in the other two groups thereafter. Consequently, the INLCD-10 group tended to have higher body weights than the CON group at the last phase of the experimental period (CON; 29.1 ± 0.6 g, INLCD-1; 30.4 ± 1.0 g vs. INLCD-10; 32.0 ± 0.7 g, p = 0.054, at experimental week 11) (Fig 2A). With respect to the energy intake, the total energy intakes from the diet were higher in the INLCD groups than in the CON group (Fig 2B).

With respect to the body composition, trunk fat mass assessed by CT scanning were significantly higher in mice consuming INLCD-10% and INLCD-1% compared to those consuming the regular diet (Table 2). In contrast, the weights of all measured muscle tissues (GC, TA, and SOL) were not affected by diet (Table 2).

**Table 2:** Body composition of mice.

	CON group	INLCD-10 group	INLCD-1 group
Trunk fat mass (g)	7.7 ± 1.3	15.8 ± 0.7 **	13.0 ± 1.0 *
Subcutaneous fat mass (g)	3.9 ± 0.7	7.1 ± 0.3 **	6.7 ± 0.5 **
Visceral fat mass (g)	3.8 ± 0.60	8.7 ± 0.4 ***	6.3 ± 0.5 **
			
Muscle weights (mg)			
Gastrocnemius muscle (GC)	140.5 ± 4.2	137.5 ± 1.9	137.0 ± 3.8
Tibialis anterior muscle (TA)	56.9 ± 3.8	55.5 ± 4.0	55.3 ± 2.2
Soleus muscle (SOL)	11.4± 0.9	11.5 ± 1.1	11.1 ± 0.4

The data are shown as mean ± SEM.

*, p < 0.05

**, p < 0.01

***, p < 0.001 vs CON.

### Effect of INLCDs on blood biochemical parameters in exercise-trained mice

There were no significant differences in blood glucose, triglyceride, or NEFA levels among the groups. In contrast, blood 3-hydroxybutyrate concentration was significantly increased in the INLCD-1 group compared to the CON group, while no significant increase was observed in the INLCD-10 group (Table 3).

**Table 3:** Blood parameters.

	CON group	INLCD-10 group	INLCD-1 group
Glucose [mg/dL]	269.1 ± 13.9	302.1 ± 20.9	295.1 ± 16.2
NEFA [mEq/L]	0.79 ± 0.06	0.69 ± 0.04	0.73 ± 0.05
Triglyceride [mg/dL]	90.7 ± 5.5	75.1 ± 4.3	78.7 ± 4.8
3-hydroxybutyrate [μmol/L]	362.4 ± 35.4	420.2 ± 57.3	558.6 ± 37.0 *

NEFA; Non-esterified fatty acid.

The data are shown as mean ± SEM.

*, p < 0.05, vs CON.

### Effects of INLCDs on muscle glycogen and endurance capacity in exercise-trained mice

**Fig 3:**
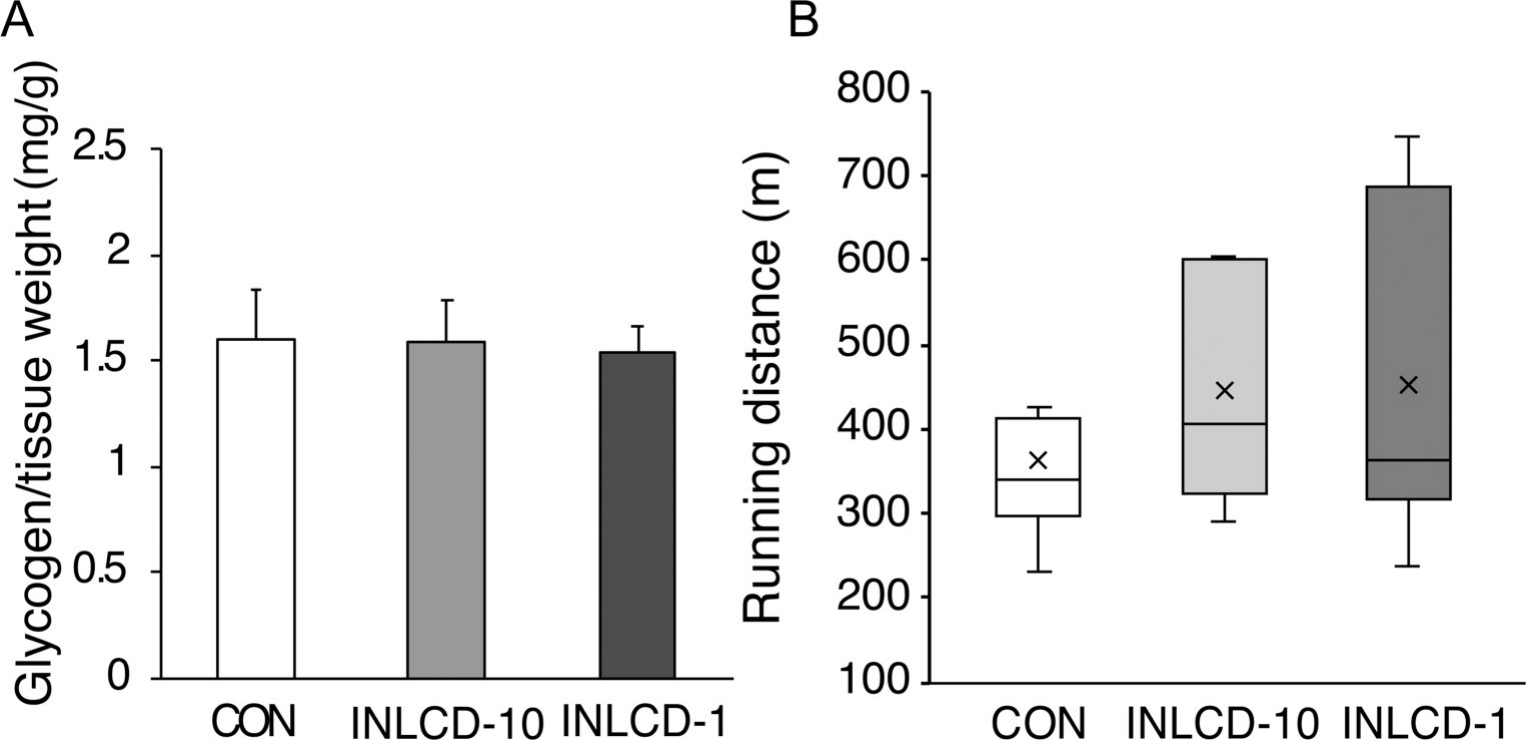
Effects of INLCD exposure on muscle glycogen content and endurance capacity in exercise-trained mice. Mice were divided into three groups: those fed the control diet (CON), those fed an INLCD-10% (INLCD-10), and those fed an INLCD-1% (INLCD-1). (A) Muscle glycogen content after experimental period in each group are shown. Data shown are means ± SEM, (n = 8 per group). (B) Endurance capacity was analyzed during week 11 of the experimental period using a treadmill. The total running distance until exhaustion are shown (n = 8 per group). Values are presented in a box-and-whisker plot; boxes are constructed with the intervals between the first and third quartiles of the data distribution; crosses and lines in the boxes are means and medians, respectively; positive and negative bars are maximum and minimum individual values in each group, respectively.

We then measured tissue glycogen content. In the end, differences in the consumed diet did not influence the muscle glycogen storage (Fig 3A). We also analyzed the endurance capacity of each mouse. Fig 3B shows the running distances till the mice reach exhaustion. The average running distances of mice in both INLCD groups did increase, but the difference in distances was not significant compared to that of the CON group.

### INLCD-induced changes in the expression of metabolism-related genes in TA muscle tissue

The expression level of genes related to energy substrate utilization was determined in TA, a glycolytic fast-twitch muscle, at the end of the 12-week experiment.

**Fig 4:**
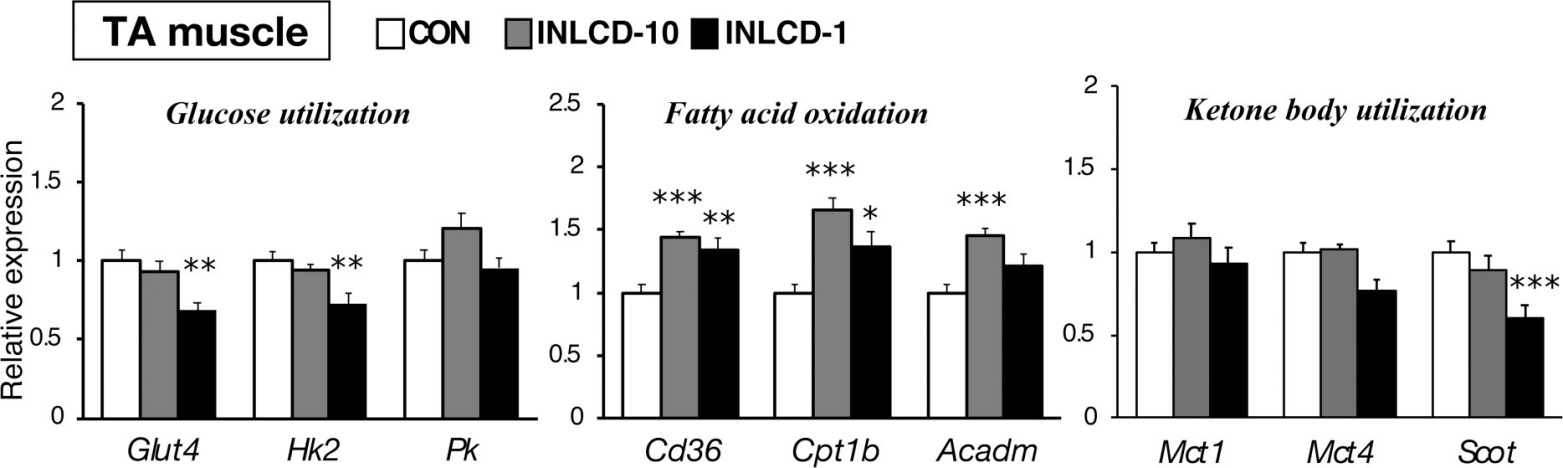
Effects of INLCD exposure on metabolic gene expression in TA muscles of mice. Gene expression levels in TA muscles of mice fed the control diet (CON, open bar), those fed an INLCD-10% (INLCD-10, gray bar), and those fed an INLCD-1% (INLCD-1, black bar) are shown. The mRNA abundance was quantified and normalized to β-actin (n = 8 per group). Data shown are means ± SEM. Values are expressed as the fold-change compared with the CON group, which was arbitrarily set to 1. *, p < 0.05; **, p < 0.01; ***, p < 0.001 vs. CON.

There was a significant decrease in the gene expression of glucose transporter-4 (*Glut4*), which is associated with glucose uptake, and of hexokinase (*Hk*), which is the main enzyme in the glycolytic pathway, in the INLCD-1 group but not in the INLCD-10 group. The gene expression of pyruvate kinase (*Pk*), which is the key enzyme in the regulation of glycolysis, did not change in INLCD groups. Both INLCD-10 and INLCD-1 groups showed significant increases in the expression of genes involved in fatty acid utilization, such as cluster of differentiation 36 (*Cd36*), which is related to fatty acid uptake, and of carnitine palmitoyltransferase 1B (*Cpt1b*), which is involved in fatty acid oxidation. Moreover, the INLCD-10 group showed further increased gene expression of medium-chain acyl-CoA dehydrogenase (*Acadm*), the main enzyme in fatty acid oxidation. Regarding ketone metabolism, the expression of succinyl-CoA:3-oxoacid CoA transferase (*Scot*), a key enzyme in ketolysis, was significantly suppressed in only the INLCD-1 group (Fig 4).

### INLCD-induced changes in the expression of metabolism-related genes in SOL muscle tissues

**Fig 5:**
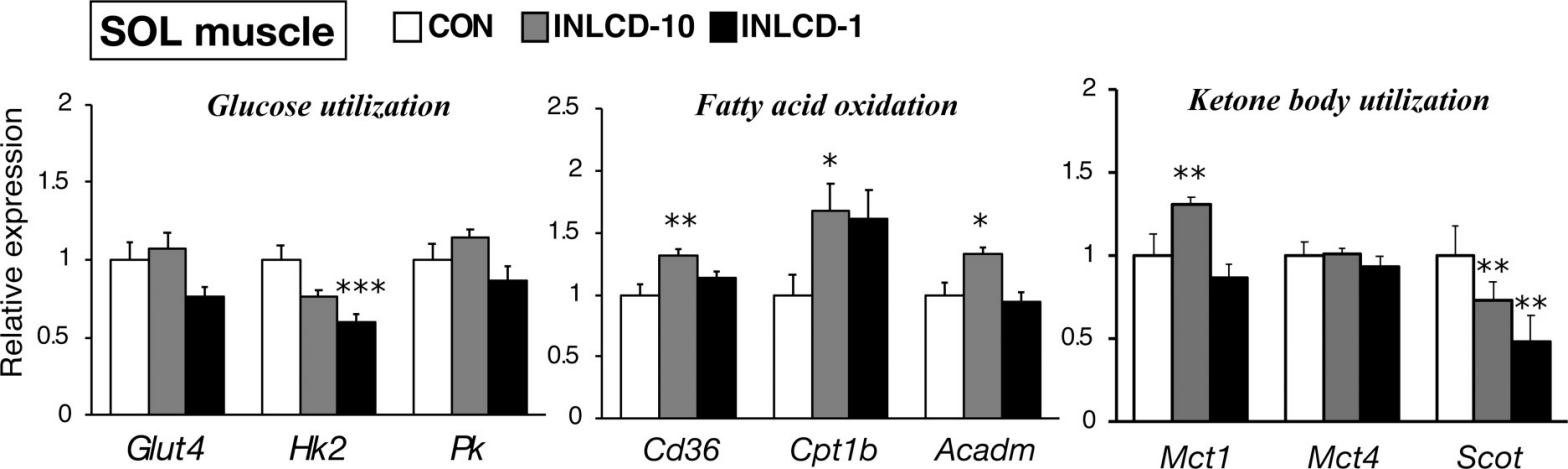
Effects of INLCD exposure on metabolic gene expression in SOL muscles of mice. Gene expression levels in the SOL muscles of mice fed the control diet (CON, open bar), those fed an INLCD-10% (INLCD-10, gray bar) and those fed an INLCD-1% (INLCD-1, black bar) groups are shown. The mRNA abundance was quantified and normalized to β-actin (n = 8 per group). Data shown are means ± SEM. Values are expressed as the fold-change compared with the CON group, which was arbitrarily set to 1. *, p < 0.05; **, p < 0.01; ***, p < 0.001 vs. CON.

We also examined the expression level of metabolic genes in SOL muscle, a representative oxidative, slow-twitch muscle. Overall, results similar to those of TA muscles were observed in SOL muscle (Fig 5), with some differences. There was a significant decrease in the expression of *Hk2* but not of *Glut 4* in the INLCD-1 group. The INLCD-10 group, but not the INLCD-1 group, showed increased expression of genes involved in fatty acid metabolism, such as *CD36*, *Acadm*, and *Cpt1b*. *Scot* expression was suppressed in the INLCD-10 and INLCD-1 groups. In contrast, monocarboxylate transporter 1 (*Mct1*) expression was only increased in the INLCD-10 group.

## Discussion

In this study, we investigated the effects of the addition of INLCD exposure to exercise training with a focus on exercise performance, muscle glycogen content, and the adaptive changes in energy metabolism at gene expression levels in skeletal muscles.

INLCD did not suppress weight gain (INLCD-1%) but rather tended to increase body weight (INLCD-10%) in C57BL/6 mice subjected to a regular exercise (p = 0.054). In humans, an LCD is a widely accepted strategy for weight loss; however, the effectiveness of a low carbohydrate intake for the management of obesity remains controversial [30, 31]. Similarly, although numerous animal studies using LCDs have been performed, the effects of LCDs on rodent body weight were inconsistent, with several studies reporting that body weight was suppressed [32–34], not changed [35, 36], or increased [37]. Moreover, several previous studies using exercise-trained animals [38, 39] have reported decreased weight in LCD-fed animals, in contrast to the present study. The tendency toward an increase in the weight of mice observed in this study may be due to the higher energy intake from the high-energy content of the tested diets. However, several previous animal studies reported that weight gain was suppressed in LCD-fed mice despite having higher energy intake than mice consuming the regular chow [32, 34].

There are several possible explanations for this discrepancy between previous studies and our study. One considerable factor might be the degree of ketosis induced by a diet. In rodents, ketosis leads to the induction of liver FGF21 by activating PPARα [40], which leads to the activation of brown adipose tissue and contributes to weight loss [41]. A previous study reported that the lesser the carbohydrate content in LCDs, the greater the weight reduction among the mice fed LCD [33], indicating that the higher degree of ketosis induced by extremely low carbohydrate content is needed for weight loss. The lower body weight in LCD-fed animals might also arise from the decreased amount of protein intake. An LCD containing less than 10% energy from protein has frequently been used in previous animal studies, and a previous study reported that a lower protein LCD induced body weight reduction, mainly due to lower lean body mass when compared to a high-protein LCD—even in an isoenergetic diet [42]. Therefore, the lack of decrease in body weight of the mice fed an INLCD in the present study might not only be due to the higher energy intake but also the isonitrogenous intake and insufficient ketosis induced by the tested diets.

Regardless of the inconsistencies in reports regarding body weight changes, previous animal studies consistently showed that consuming LCD increased fat mass in rodents, as observed in the present study, and reduced their lean body mass [33, 35, 37, 43]. In contrast to our study, in certain human studies, significant decreases in the body fat mass have been observed in athletes or in trained subjects adhering to an LCD regimen with regular exercise [44–46]. Disparities have been reported to exist between rodent and human adiposity in terms of adipose tissue development and distribution [47]; thus, it could be a possibility that fat deposition occurs easily in rodents, when compared to humans, upon consumption of high-fat LCDs. Regarding the lean body mass, although total lean body mass could not be determined in this study because CT imaging was performed only on the body trunk area, the weight of each muscle in the hind limbs did not decline following INLCD consumption. Consistent with the present result, a previous animal experiment showed that the lean body mass was maintained with an LCD containing 19.1% protein but not with one containing 5.5% protein [33]. Thus, an adequate protein intake is important to prevent loss in lean body mass while adopting an LCD.

Consistent with a classical human study [48] and our previous study [22], no changes were observed in the concentration of serum glucose and NEFA between INLCD-fed mice and control mice. The blood 3-hydroxybutyrate acid levels of the mice significantly increased in the INLCD-1 group. Normally, when consuming an LCD, excess ketone bodies are produced by the liver and then carried to other tissues by the blood to be used as an energy source [49, 50]. Additionally, ketone bodies such as 3-hydroxybutyrate act as a signaling molecule that regulates physiological functions, such as energy metabolism and stress control [51, 52]. From this perspective, an increase in blood ketone body concentration in INLCD-1 groups might cause specific changes in gene expression that would control energy metabolism.

We assessed the effect of INLCDs on muscle glycogen storage using gastrocnemius muscles, which has often been used for glycogen measurement in rodent studies. Our results showed that glycogen storage in the muscles did not differ among the three groups. The effects of low carbohydrate consumption on muscle glycogen storage have been hardly studied in trained animals but widely studied in athletes. However, the results are inconsistent. Webster et al. [53] reported significantly less muscle glycogen in cyclists consuming an LCD (7% carbohydrate, 72% fat, 21% protein) for more than 8 months compared to subjects consuming a regular diet. In contrast, Volek et al. [54] reported that consuming an LCD (10% carbohydrate, 70% fat, 20% protein) for an average of 20 months did not decrease the muscle glycogen compared to a traditional high carbohydrate (59% carbohydrate, 25% fat, 15% protein) diet in ultra-endurance runners. We previously reported that glycogen storage significantly decreased in the muscles of sedentary LCD-fed mice but not in exercise-training animals [22]. Considering the results of the current study that revealed an increasing tendency in the expression levels of genes related to fatty acid oxidation, the combination of regular exercise and INLCD consumption might enhance the preferential utilization of fatty acids and eliminate the shortage of intra-muscular glucose storage induced by LCD consumption.

In the present study, the endurance capacity of exercise-trained mice was not changed by the INLCD. In contrast, a recent report investigating the effect of LCD on exercise capacity using the same strain of animals reported that an 8-week LCD enhanced the exhaustive exercise capacity of the mice [19]. This inconsistency could possibly be explained by the difference in training status of the animals (trained/untrained) or in the composition of LCD diets between the studies. In fact, regarding the efficacy of the LCD for performance benefits, a recent review addressed the heterogeneous consequences among studies on endurance athletes, in which the macronutrient composition of tested LCDs varied between studies [13, 55]. Further research focusing on subtle differences in nutrient composition is needed to better understand the effects of LCD consumption on endurance performance.

Fast-twitch and slow-twitch muscles differ at the level of energy metabolism. For example, fast-twitch fibers typically display lower capacity for oxidative metabolism and preferentially use the glycolytic pathway. We therefore assessed gene expression separately in TA and SOL muscles, the representative fast-twitch and slow-twitch muscles, respectively. We found that INLCD consumption caused specific changes in metabolic gene expression in a similar pattern in both TA and SOL. INLCD-1% caused significant decreases in the expression of glucose utilization-related genes, *Glut4* in TA and *Hk2* in TA and SOL, while these decreases were not induced by INLCD-10%. Previous research showed that low carbohydrate intake leads to the suppression of insulin-induced glucose uptake by skeletal muscles [56], which may be a protective phenomenon to prevent a decline in blood glucose concentration. Similarly, our results suggest that extreme restriction, but not moderate restriction, of carbohydrate in an INLCD may cause the suppression of glucose utilization in muscles, such that even gene expression levels are affected, to avoid a decline in blood glucose concentration.

Considering fatty acid metabolism, investigations of genes involved in lipid oxidation performed in both humans and animal models have shown that consuming a low carbohydrate/high-fat diet increases lipid oxidation-related genes, such as *Cd36* and *Cpt1*, in skeletal muscles [57–59]. Our results showed that INLCDs, even INLCD-10% that induced no significant ketosis, enhanced the expression of these genes in exercise-trained mice. A significant decrease in the respiratory exchange ratio following intervention with LCD has been reported previously in rodents [18, 60], and a recent meta-analysis has clearly demonstrated that this occurs in athletes following LCD intervention as well, indicating an enhanced ability to oxidize fats [55]. These increases in ability could be caused, at least in part, by the increases in the expression of lipid oxidation-related genes in muscle tissues induced by INLCDs.

Ketone bodies also serve as an alternative fuel source, and SCOT is an enzyme required to utilize ketone bodies as an energy source. In this study, *Scot* expression was reduced in both TA and SOL muscles by INLCD consumption, and this reduction appeared to correlate with the carbohydrate content of the INLCDs. Similarly, Wentz et al. [61] reported that *Scot* expression was decreased in the hearts of mice fed an LCD. Although inhibiting the use of ketone bodies during the LCD treatment may appear counterintuitive, ketone bodies are the only alternative brain energy source for glucose; this may explain why the heart and skeletal muscles suppress *Scot* expression to ensure sufficient ketone bodies for the brain when dietary carbohydrates are insufficient.

**Fig 6:**
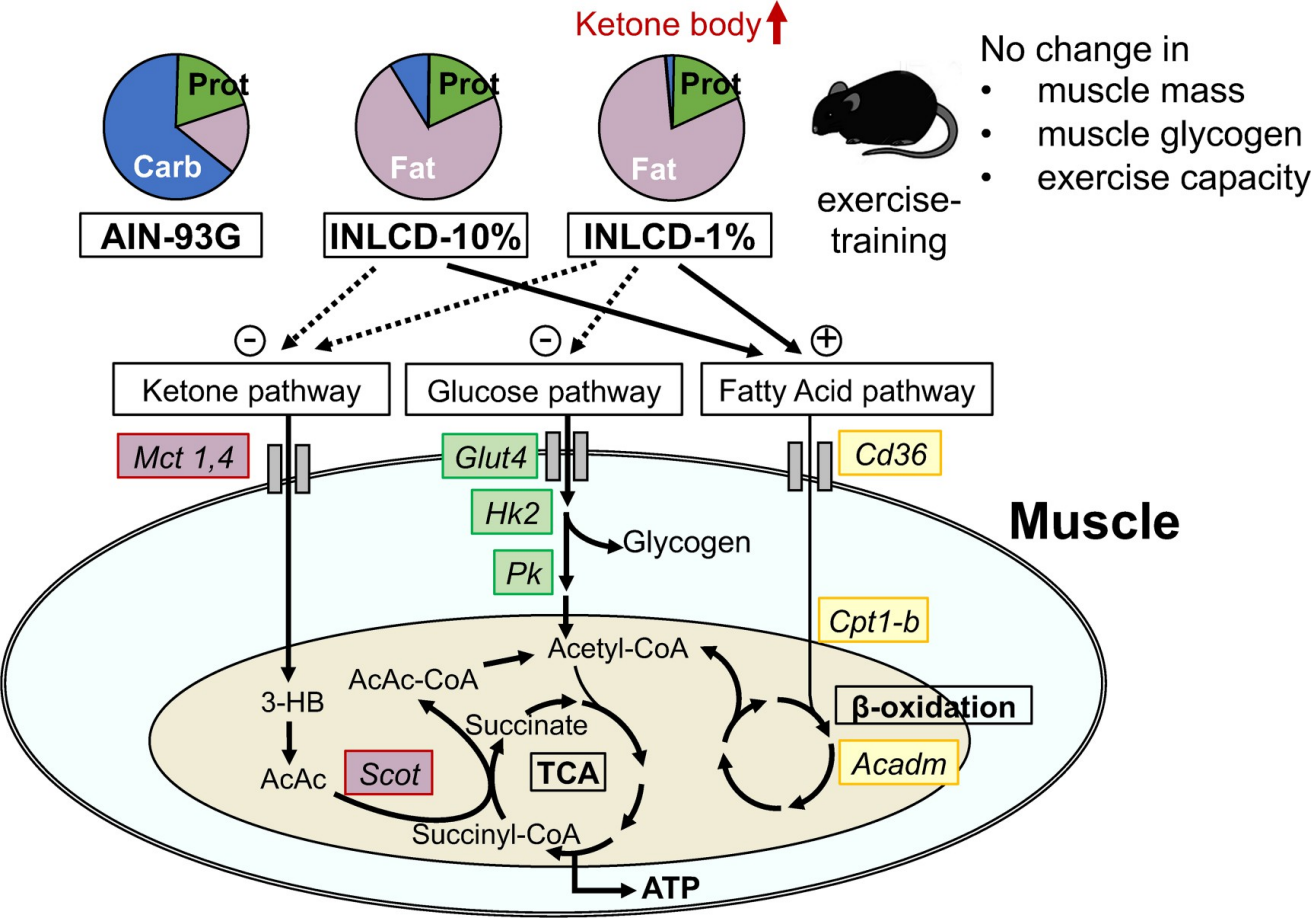
The effects of isonitrogenous INLCDs on exercise-trained mice. Carb; carbohydrate, Prot; protein, *Mct*; monocarboxylic acid transporter, *Glut4*; glucose transporter 4, *Hk2*; hexokinase 2, *Pk*; pyruvate kinase, *Cd36*; cluster of differentiation 36, *Cpt1b*; carnitine palmitoyltransferase 1b, *Acadm*; medium-chain acyl-CoA dehydrogenase, *Scot*; succinyl-CoA 3-oxoacid CoA transferase, 3-HB; 3-hydroxybutyrate, AcAc; acetoacetic acid.

Finally, a summary of the findings of this study is shown in Fig 6.

## Conclusion

In conclusion, both INLCDs with 1% and 10% carbohydrate content, respectively, and a protein content adjusted to that of a regular rodent diet serving as a control, did not suppress weight gain but maintained muscle mass. Furthermore, these diets did not exert any effect on the exercise capacity or the muscle glycogen content of trained mice. However, these diets still had a specific impact on metabolic gene expression in muscles based on carbohydrate content. Intriguingly, INLCD-10% seemed to affect the expression of lipid oxidation-related genes more compared to INLCD-1% despite no significant ketosis, although the exact mechanism by which INLCD-10% induced a higher increase in oxidative metabolism at the genetic level than INLCD-1% remains unclear. To determine how the carbohydrate/fat/protein ratio in an LCD affects the expression of metabolic genes in muscular tissues, additional studies would be needed using LCDs in which various ratios of components are tested.

## Supporting information

S1 Table Primer sequences for the quantitative PCR. (DOCX)
