# Supplementary material for: Isonitrogenous low-carbohydrate diet elicits specific changes in metabolic gene expression in the skeletal muscle of exercise-trained mice
Source: PLoS One. 2022 Jan 21;17(1):e0262875. doi: 10.1371/journal.pone.0262875 (PMC8782354; doi:10.1371/journal.pone.0262875)
Supplement: S1 Table — (DOCX) [file pone.0262875.s001.docx]

Table S1.　 Primer sequences for the quantitative PCR

|  | Forward | Reverse |
| --- | --- | --- |
| *Glut4* | GACGGACACTCCATCTGTTG | CATAGCTCATGGCTGGAACC |
| *Pk* | CGATCTGTGGAGATGCTGAA | AATGGGATCAGATGCAAAGC |
| *Hk2* | GAAGGGGCTAGGAGCTACCA | CTCGGAGCACACGGAAGTT |
| *Cd36* | TGGCCTTACTTGGGATTGG | CCAGTGTATATGTAGGCTCATCCA |
| *Cpt1b* | GCACACCAGGCAGTAGCTTT | CAGGAGTTGATTCCAGACAGGTA |
| *Acadm* | ACTGACGCCGTTCAGATTTT | GCTTAGTTACACGAGGGTGATG |
| *Mct1* | GTGACCATTGTGGAATGCTG | CTCCGCTTTCTGGCCACATCTC |
| *Mct4* | GGCTGTTTTATCATCACGGGTT | GTGTCGCTGTAGCCATTCCC |
| *Scot* | TGGCCAACTGGATGATACCTGG | TCCATGGTGACCACCACTTTGG |
| *β-actin* | CTAAGGCCAACCGTGAAAAG | ACCAGAGGCATACAGGGACA |

*Glut4* : glucose transporter 4

*Pk* : pyruvate kinase

*Hk2* : hexokinase 2

*Cd36* : cluster of differentiation 36

*Cpt1b* : carnitine palmitoyltransferase1-b

*Acadm* : medium-chain acyl-CoA dehydrogenase

*Mct1* : monocarboxylic acid transporter 1

*Mct4* : monocarboxylic acid transporter 4

*Scot* : Succinyl CoA 3-oxoacid CoA transferase
